# Supplementary material for: Contraceptive uptake and characteristics in the immediate postpartum period among women attending Tygerberg Academic Hospital
Source: PLoS One. 2026 May 28;21(5):e0349790. doi: 10.1371/journal.pone.0349790 (PMC13218452; doi:10.1371/journal.pone.0349790)
Supplement: Attachment — Submitted filename: Response to Reviewers.docx [file pone.0349790.s002.docx]

Dear Editor in Chief,

Thank you for the opportunity to re-submit our article to PLOS ONE journal. Please find below a rebuttal table outlining the changes and revisions requested for our submission.

| Changes requested | Revision action. |
| --- | --- |
| Title: Authors should remove the academic qualifications from the authors' list The title needs review. "A cross-sectional prevalence study demonstrating a high uptake of postpartum family planning in a high risk setting in South Africa." what "high risk" are the authors referring to? HIV prevalence? poor contraceptive uptake? etc. I suggest: "Prevalence and sociodemographic risk and morbidities associated with contraceptive uptake among postpartum women at ...... A cross-sectional study". This is a suggestion that can be reviewed by the authors. | The title has been revised and edited accordingly. The authors academic qualifications have been removed. |
| Authors should use continuous number line when submitting for efficient review | Continuous number line have been inserted. |
| PLOS requires an ORCID iD for the corresponding author in Editorial Manager on papers submitted after December 6th, 2016. Please ensure that you have an ORCID iD and that it is validated in Editorial Manager. To do this, go to ‘Update my Information’ (in the upper left-hand corner of the main menu), and click on the Fetch/Validate link next to the ORCID field. This will take you to the ORCID site and allow you to create a new iD or authenticate a pre-existing iD in Editorial Manager. | Orchid numbers have been added and updated.  Denise Sproul: [0009-0003-2138-0495](https://orcid.org/0009-0003-2138-0495)  Gerhard Hanekom: 0000-0002-2832-9436  Michael McCaul: [0000-0002-2730-6478](https://orcid.org/0000-0002-2730-6478) |
| Thank you for stating the following financial disclosure: We would like to acknowledge the Temporary Research funding support from Stellenbosch University’s Subcommittee C of the Research Committee. As well as the funding received from the GELA project which is part of the EDCTP2 programme supported by the European Union (grant number RI2020S-3303 - GELA). The views and opinions of authors expressed herein do not necessarily state or reflect those of EDCTP.    Please state what role the funders took in the study.  If the funders had no role, please state: ""The funders had no role in study design, data collection and analysis, decision to publish, or preparation of the manuscript.""  If this statement is not correct you must amend it as needed.  Please include this amended Role of Funder statement in your cover letter; we will change the online submission form on your behalf | All related revisions have been updated, and additional statement has been added to the manuscript and cover letter. |
| TYPOGRAPHICAL ERRORS Pg 8: delete full stop at the end of the title Pg 14: (Statistical Analysis) correction “confident interval” for “confidence interval” Pg 15: (Results) correction “summarized” for “summarized” consistency in British spelling | All correctios have been made. |
| 3. Authors should read through the authors guideline and adhere strictly to it. | We have made adjustments accordingly. |
| 4. Authors should add more numbers to the statistical result of the abstract. | More results have been added to the abstract section and shown in red. |
| 5.Please remove the Table in the introduction | The table has been removed as requested. |
| 6.The statistical analysis did not describe a rigorous statistical analysis that is of quality for publishing in Plos one. I strongly recommend that authors should conduct bivariate analysis and if possible appropriate regression modelling to before the manuscript with be of high quality that can be published in PLOS ONE. The sample size is adequate for useful conclusion from the study | A bivariant analysis and multivariant analysis have been conducted and findings have been added to relevant sections. |
| Authors stated .".....standard deviation (SD), and range for continuous variables if normally distributed" What about if the continuous variable was not normally distributed? | This has been revised and reviewed in the manuscript. If not normally distributed interquartile ranges and medians have been used. |
| In the Result section, authors stated "Over a period of seven months 1066 participants were recruited for this study and had a recorded family planning method of their choice."  From the methodology section, it appears that the authors described retrospective chart review of routinely collected data. Please authors should be explicit about what study was conducted. was it a retrospective or prospective study? | A prospective study was conducted. This has been revised in the manuscript. |
| Table 1 is not looking "professional" For example: "Delivery Characteristics" lumped many unrelated categories. One of the variables in that category is mode of delivery that can be vaginal delivery, operative vaginal delivery or caesarean section. Birth outcome such as live birth, fresh still birth, early neonatal death etc may be another category etc, | The tables have been split and edited as requested into table 2 and 3. |
| This study seems to be among postpartum women. When after delivery was the interview conducted.? If it was anytime post partum. It will be good to know how soon after delivery that women presented for family planning at the hospitals. | The data was collected from the medical records on the day of discharge after delivery. This has been stated in the methods section as well. |
| Table 2.  gravidity and parity are not likely to be normally distributed. Thus median and IQR may be more appropriate. Please check | These statistics have been re-analysed and updated accordingly. |
| Discussion: Authors to cite relevant literatures as required | Revisions done. |
| Please ensure that DMPA had been previously defined in the manuscript | DMPA has been defined clearly in the text. |
| Are there any historical and structural issues that influences the contraceptive uptake pattern? | Additional paragraph added to discussion as requested. |
| Social cultural and economic determinants of health is a very important issue in South Africa. However, authors did not describe the ethnic characteristics of the studied participants. Occupation and income of the participants are also important information to provide. | This revision is addressed and discussed in the discussion section. |
| If the prevalence is so high, is it possible to collect further data for more months to assist to obtain more participants that will not take the contraceptive for easy comparative cross-sectional study? | This limitation is addressed in the limitations section. |
| What are the reasons for the comparatively high contraceptive uptake in this population as compared with other south African and global studies? | Revised and discussed in the discussion section. |
| Authors stated "There is also a theoretical risk that high dose progesterone exposure may impact the development of the infant brain. Authors should cite appropriate reference for this statement | The WHO statement that makes this claim is cited as well as the original data. |

All revisions and changes have also been marked in the text using red font and comment boxes.

Kind regards

Denise
