# Supplementary material for: Contraceptive uptake and characteristics in the immediate postpartum period among women attending Tygerberg Academic Hospital
Source: PLoS One. 2026 May 28;21(5):e0349790. doi: 10.1371/journal.pone.0349790 (PMC13218452; doi:10.1371/journal.pone.0349790)
Supplement: Attachment — Submitted filename: PONE-D-24-39098_R1.docx [file pone.0349790.s003.docx]

PLOS ONE

Prevalence and sociodemographic risk and morbidities associated with contraceptive uptake among postpartum women at Tygerberg Academic Hospital, Cape Town: A cross-sectional study.

--Manuscript Draft--

| Manuscript Number: | PONE-D-24-39098R1 |
| --- | --- |
| Article Type: | Research Article |
| Full Title: | Prevalence and sociodemographic risk and morbidities associated with contraceptive uptake among postpartum women at Tygerberg Academic Hospital, Cape Town: A cross-sectional study. |
| Short Title: | High prevalence of postpartum family planning found in South Africa. |
| Corresponding Author: | Denise Sproul, M.D, MMed, MSc.  Stellenbosch University - Tygerberg Campus: Stellenbosch University Faculty of  Medicine and Health Sciences  Cape Town, Western Cape SOUTH AFRICA |
| Keywords: | family planning; contraception; postpartum; pregnancy interval spacing; Prevalence. |
| Abstract: | The immediate postpartum period is a unique opportunity to motivate and educate women on family planning options. This study aimed to determine the prevalence of family planning use in our setting. Secondary objectives include determining the frequency of the various methods used, safety of the method, common risk factors, the interval spacing between births and associations with family planning method choices. A cross-sectional study was conducted at Tygerberg Academic Hospital, South Africa, which serves a predominantly low socioeconomic status and high-risk obstetric population. Medical records were assessed prospectively from January 2023 till July 2023. Data was collected and captured from maternity medical records on the day of discharge. The statistical analysis was done using frequencies and percentages, with 95% confidence intervals (95%CI). A secondary bivariant analysis was done to evaluate the associations between these variables. Of the 1066 participants included, 97.5% (95%CI 96.5-98.5) received family planning. Majority of the patients (48.8%) requested the injectable depo medroxyprogesterone acetate. These patients who chose this method were on average younger (OR 0.962, p<0.001), had a lower BMI (OR 0.983, p=0.012), and are a higher parity (OR 0.721, p<0.001). HIV positive women were also 35% less likely (OR 0.651, p= 0.008) to choose this method compared to other methods. Of the 1039 patients who had received family planning, 47.8% were classified into a World Health Organization medical eligibility category three. This was almost exclusively attributed due to patients who were breastfeeding and requested the depo medroxyprogesterone acetate as their family planning method postpartum. Majority (66%) of the patients had spaced their pregnancies more than two years apart. The exceptionally high prevalence of uptake of family planning indicates it can be successfully implemented in the postpartum period. Patients should be counselled individually on the safest and most appropriate method. |
| Order of Authors: | Denise Sproul, M.D, MMed, MSc. |
|  | Gerhard Hanekom, M.D. MMed, MPhil |
|  | Michael McCaul, M.D. MMed, MPhil |
| Response to Reviewers: | Changes requestedRevision action.  Title: Authors should remove the academic qualifications from the authors' list The title needs review. "A cross-sectional prevalence study demonstrating a high uptake of postpartum family planning in a high risk setting in South Africa." what "high risk" are the authors referring to? HIV prevalence? poor contraceptive uptake? etc. I suggest: "Prevalence and sociodemographic risk and morbidities associated with contraceptive uptake among postpartum women at ...... A cross-sectional study". This is a suggestion that can be reviewed by the authors.The title has been revised and edited accordingly.  The authors academic qualifications have been removed.  Authors should use continuous number line when submitting for efficient reviewContinuous number line have been inserted. |

|  | PLOS requires an ORCID iD for the corresponding author in Editorial Manager on papers submitted after December 6th, 2016. Please ensure that you have an ORCID iD and that it is validated in Editorial Manager. To do this, go to ‘Update my Information’ (in the upper left-hand corner of the main menu), and click on the Fetch/Validate link next to the ORCID field. This will take you to the ORCID site and allow you to create a new iD or authenticate a pre-existing iD in Editorial Manager.Orchid numbers have been added and updated.  Denise Sproul: 0009-0003-2138-0495  Gerhard Hanekom: 0000-0002-2832-9436  Michael McCaul: 0000-0002-2730-6478  Thank you for stating the following financial disclosure: We would like to acknowledge the Temporary Research funding support from Stellenbosch University’s  Subcommittee C of the Research Committee. As well as the funding received from the  GELA project which is part of the EDCTP2 programme supported by the European Union (grant number RI2020S-3303 - GELA). The views and opinions of authors expressed herein do not necessarily state or reflect those of EDCTP.  Please state what role the funders took in the study. If the funders had no role, please state: ""The funders had no role in study design, data collection and analysis, decision to publish, or preparation of the manuscript.""  If this statement is not correct you must amend it as needed.  Please include this amended Role of Funder statement in your cover letter; we will change the online submission form on your behalfAll related revisions have been updated, and additional statement has been added to the manuscript and cover letter.  TYPOGRAPHICAL ERRORS  Pg 8: delete full stop at the end of the title  Pg 14: (Statistical Analysis) correction “confident interval” for “confidence interval” Pg 15: (Results) correction “summarized” for “summarized” consistency in British spelling  All correctios have been made.   3. Authors should read through the authors guideline and adhere strictly to it. We havemade adjustments accordingly. 4. Authors should add more numbers to the statistical result of the abstract.Moreresults have been added to the abstract section and shown in red.   5.Please remove the Table in the introductionThe table has been removed as requested.  6.The statistical analysis did not describe a rigorous statistical analysis that is of quality for publishing in Plos one. I strongly recommend that authors should conduct bivariate analysis and if possible appropriate regression modelling to before the manuscript with be of high quality that can be published in PLOS ONE. The sample size is adequate for useful conclusion from the studyA bivariant analysis and multivariant analysis have been conducted and findings have been added to relevant sections.  Authors stated .".....standard deviation (SD), and range for continuous variables if normally distributed" What about if the continuous variable was not normally distributed?This has been revised and reviewed in the manuscript. If not normally distributed interquartile ranges and medians have been used.  In the Result section, authors stated "Over a period of seven months 1066 participants were recruited for this study and had a recorded family planning method of their choice." From the methodology section, it appears that the authors described retrospective chart review of routinely collected data. Please authors should be explicit about what study was conducted. was it a retrospective or prospective study?A prospective study was conducted. This has been revised in the manuscript.  Table 1 is not looking "professional" For example: "Delivery Characteristics" lumped many unrelated categories. One of the variables in that category is mode of delivery that can be vaginal delivery, operative vaginal delivery or caesarean section. Birth outcome such as live birth, fresh still birth, early neonatal death etc may be another category etc,The tables have been split and edited as requested into table 2 and 3. This study seems to be among postpartum women. When after delivery was the interview conducted.? If it was anytime post partum. It will be good to know how soon after delivery that women presented for family planning at the hospitals. The data was collected from the medical records on the day of discharge after delivery. This has been stated in the methods section as well.  Table 2. gravidity and parity are not likely to be normally distributed. Thus median and  IQR may be more appropriate. Please checkThese statistics have been re-analysed |
| --- | --- |

|  | and updated accordingly.  Discussion: Authors to cite relevant literatures as requiredRevisions done.  Please ensure that DMPA had been previously defined in the manuscriptDMPA has been defined clearly in the text.  Are there any historical and structural issues that influences the contraceptive uptake pattern? Additional paragraph added to discussion as requested.  Social cultural and economic determinants of health is a very important issue in South Africa. However, authors did not describe the ethnic characteristics of the studied participants. Occupation and income of the participants are also important information to provide. This revision is addressed and discussed in the discussion section. If the prevalence is so high, is it possible to collect further data for more months to assist to obtain more participants that will not take the contraceptive for easy comparative cross-sectional study?This limitation is addressed in the limitations section.  What are the reasons for the comparatively high contraceptive uptake in this population as compared with other south African and global studies?Revised and discussed in the discussion section.  Authors stated "There is also a theoretical risk that high dose progesterone exposure may impact the development of the infant brain. Authors should cite appropriate reference for this statementThe WHO statement that makes this claim is cited as well as the original data. |
| --- | --- |
| Additional Information: |  |
| Question | Response |
| Financial Disclosure  Enter a financial disclosure statement that describes the sources of funding for the work included in this submission. Review the [submission guidelines](http://journals.plos.org/plosone/s/submission-guidelines#loc-financial-disclosure-statement) for detailed requirements. View published research articles from [PLOS ONE](https://journals.plos.org/plosone/) for specific examples.  This statement is required for submission and will appear in the published article if the submission is accepted. Please make sure it is accurate. | Yes |
| Funded studies  Enter a statement with the following details:   - Initials of the authors who received each award - Grant numbers awarded to each author - The full name of each funder - URL of each funder website - Did the sponsors or funders play any role in the study design, data collection and analysis, decision to publish, or preparation of the manuscript? |  |
| Did you receive funding for this work? |  |

| Please add funding details.   as follow-up to "Financial Disclosure  Enter a financial disclosure statement that describes the sources of funding for the work included in this submission. Review the [submission guidelines](http://journals.plos.org/plosone/s/submission-guidelines#loc-financial-disclosure-statement) for detailed requirements. View published research articles from [PLOS ONE](https://journals.plos.org/plosone/) for specific examples.  This statement is required for submission and will appear in the published article if the submission is accepted. Please make sure it is accurate. | We would like to acknowledge the Temporary Research funding support from  Stellenbosch University’s Subcommittee C of the Research Committee. As well as the funding received from the GELA project which is part of the EDCTP2 programme supported by the European Union (grant number RI2020S-3303 - GELA). The views and opinions of authors expressed herein do not necessarily state or reflect those of EDCTP. The funders had no role in study design, data collection and analysis, decision to publish, or preparation of the manuscript. |
| --- | --- |
| Funded studies  Enter a statement with the following details:   - Initials of the authors who received each award - Grant numbers awarded to each author - The full name of each funder - URL of each funder website - Did the sponsors or funders play any role in the study design, data collection and analysis, decision to publish, or preparation of the manuscript? |  |
| Did you receive funding for this work?" |  |
| Please select the country of your main research funder (please select carefully as in some cases this is used in fee calculation).   as follow-up to "Financial Disclosure  Enter a financial disclosure statement that describes the sources of funding for the work included in this submission. Review the [submission guidelines](http://journals.plos.org/plosone/s/submission-guidelines#loc-financial-disclosure-statement) for detailed requirements. View published research articles from [PLOS ONE](https://journals.plos.org/plosone/) for specific examples.  This statement is required for submission and will appear in the published article if the submission is accepted. Please make sure it is accurate. | SOUTH AFRICA - ZA |

| Funded studies  Enter a statement with the following details:   - Initials of the authors who received each award - Grant numbers awarded to each author - The full name of each funder - URL of each funder website - Did the sponsors or funders play any role in the study design, data collection and analysis, decision to publish, or preparation of the manuscript? |  |
| --- | --- |
| Did you receive funding for this work?" |  |
| Competing Interests  Use the instructions below to enter a competing interest statement for this submission. On behalf of all authors, disclose any [competing interests](http://journals.plos.org/plosone/s/competing-interests) that could be perceived to bias this work—acknowledging all financial support and any other relevant financial or nonfinancial competing interests.  This statement is required for submission and will appear in the published article if the submission is accepted. Please make sure it is accurate and that any funding sources listed in your Funding Information later in the submission form are also declared in your Financial Disclosure statement.  View published research articles from [PLOS ONE](http://journals.plos.org/plosone/) for specific examples. | The authors have declared that no competing interests exist. |

| NO authors have competing interests  Enter: The authors have declared that no competing interests exist.  Authors with competing interests  Enter competing interest details beginning with this statement:  I have read the journal's policy and the authors of this manuscript have the following competing interests: [insert competing interests here] |  |
| --- | --- |
| * typeset |  |
| Ethics Statement  Enter an ethics statement for this submission. This statement is required if the study involved:   - Human participants - Human specimens or tissue - Vertebrate animals or cephalopods - Vertebrate embryos or tissues - Field research   Write "N/A" if the submission does not require an ethics statement.  General guidance is provided below. Consult the [submission guidelines](http://journals.plos.org/plosone/s/submission-guidelines#loc-guidelines-for-specific-study-types) for detailed instructions. Make sure that all information entered here is included in the Methods section of the manuscript. | Ethical approval was obtained from the Health Research Ethics Committee of Stellenbosch University (S22/09/188), as well as the Western Cape Government for access to records and patient files. Informed consent was waivered, as only medical records were used for data collection with no patient interaction. Waiver of informed consent was approved by the ethics committee as no patient interaction occurred. The department of Western Cape government acted as a proxy for permission to the medical records, and permission was granted. |

| Format for specific study types  Human Subject Research (involving human participants and/or tissue)   - Give the name of the institutional review board or ethics committee that approved the study - Include the approval number and/or a statement indicating approval of this research - Indicate the form of consent obtained (written/oral) or the reason that consent was not obtained (e.g. the data were analyzed anonymously)   Animal Research (involving vertebrate animals, embryos or tissues)   - Provide the name of the Institutional Animal Care and Use Committee (IACUC) or other relevant ethics board that reviewed the study protocol, and indicate whether they approved this research or granted a formal waiver of ethical approval - Include an approval number if one was obtained - If the study involved non-human primates, add additional details about animal welfare and steps taken to ameliorate suffering - If anesthesia, euthanasia, or any kind of animal sacrifice is part of the study, include briefly which substances and/or methods were applied   Field Research  Include the following details if this study involves the collection of plant, animal, or other materials from a natural setting:   - Field permit number - Name of the institution or relevant body that granted permission |  |
| --- | --- |
|  |  |
| Data Availability  Authors are required to make all data underlying the findings described fully available, without restriction, and from the time of publication. PLOS allows rare exceptions to address legal and ethical concerns. See the [PLOS Data Policy](http://journals.plos.org/plosone/s/data-availability) and [FAQ](http://journals.plos.org/plosone/s/data-availability#loc-faqs-for-data-policy) for detailed information. | Yes - all data are fully available without restriction |

| A Data Availability Statement describing where the data can be found is required at submission. Your answers to this question constitute the Data Availability Statement and will be published in the article, if accepted. |  |
| --- | --- |
| Important: Stating ‘data available on request from the author’ is not sufficient. If your data are only available upon request, select ‘No’ for the first question and explain your exceptional situation in the text box. |  |
| Do the authors confirm that all data underlying the findings described in their manuscript are fully available without restriction? |  |
| Describe where the data may be found in full sentences. If you are copying our sample text, replace any instances of XXX with the appropriate details.   - If the data are held or will be held in a public repository, include URLs, accession numbers or DOIs. If this information will only be available after acceptance, indicate this by ticking the box below. For example: All XXX files   are available from the XXX database (accession number(s) XXX, XXX.).   - If the data are all contained within the manuscript and/or Supporting Information files, enter the following: All relevant data are within the manuscript and its Supporting Information files. - If neither of these applies but you are able to provide details of access elsewhere, with or without limitations, please do so. For example:   Data cannot be shared publicly because of [XXX]. Data are available from the XXX Institutional Data Access / Ethics Committee (contact via XXX) for researchers who meet the criteria for access to confidential data.  The data underlying the results presented in the study are available from (include the name of the third party | All relevant data are within the manuscript and its Supporting Information files. (Excel spread sheet, tables and interpretation) |

| and contact information or URL).  • This text is appropriate if the data are owned by a third party and authors do not have permission to share the data.  * typeset |  |
| --- | --- |
| Additional data availability information: |  |

Manuscript	[Click here to access/download;Manuscript;Manuscript.docx](https://www2.cloud.editorialmanager.com/pone/download.aspx?id=39898594&guid=b361db16-859c-42d8-bc29-f40eded50e34&scheme=1)

1 **Prevalence and sociodemographic risk and morbidities associated**
2 **with contraceptive uptake among postpartum women at Tygerberg**
3 **Academic Hospital, Cape Town: A cross-sectional study.**
4 Denise Sproul^1,2^, Michael McCaul^1^, Gerhard Hanekom^2^.

5

6 ^1^Divison of Epidemiology and Biostatistics, Department of Global Health,
7 Stellenbosch University, Cape Town, South Africa.
8 ^2^Department of Obstetrics and Gynaecology, Stellenbosch University, Cape Town, 9	South Africa.

10

11**Corresponding author:**
12 Denise Sproul.
13 Department of Obstetrics and Gynaecology, Stellenbosch University, 14	Francie Van Zyl Drive, Cape Town, South Africa.

15	Email: dsproul@sun.ac.za 16	**ORCHID iD:**

17 Denise Sproul: [0009-0003-2138-0495](https://orcid.org/0009-0003-2138-0495)
18 Gerhard Hanekom: 0000-0002-2832-9436
19 Michael McCaul: [0000-0002-2730-6478](https://orcid.org/0000-0002-2730-6478)

20

21 **Short Title:**
22 **High prevalence of postpartum family planning found in South Africa.**

# Abstract

The immediate postpartum period is a unique opportunity to motivate and educate women on family planning options. This study aimed to determine the prevalence of family planning use in our setting. Secondary objectives include determining the frequency of the various methods used, the safety of the method(s), common risk factors, the interval spacing between births and associations with family planning method choices. A cross-sectional study was conducted at Tygerberg Academic Hospital, South Africa, which serves a predominantly low socioeconomic status and high-risk obstetric population. Medical records were assessed prospectively from January 2023 till July 2023. Data was collected and captured from maternity medical records on discharge. The statistical analysis was done using frequencies and percentages, with 95% confidence intervals (95%CI). A secondary bivariant analysis was done to evaluate the associations between these variables. Of the 1066 participants included, 97.5% (95%CI 96.5-98.5) received family planning. Majority of the patients (48.8%) requested the injectable depo medroxyprogesterone acetate. These patients who chose this method were on average younger (OR 0.962, p<0.001), had a lower BMI (OR 0.983, p=0.012), and are a higher parity (OR 0.721, p<0.001). HIV positive women were also 35% less likely (OR 0.651, p= 0.008) to choose this method compared to other methods. Of the 1039 patients who had received family planning, 47.8% were classified into a World Health Organization medical eligibility category three. This was almost exclusively attributed due to patients who were breastfeeding and requested the depo medroxyprogesterone acetate as their family planning method postpartum. Majority (66%) of the patients had spaced their pregnancies more than two years apart. The exceptionally high

48 prevalence of uptake of family planning indicates it can be successfully implemented
49 in the postpartum period. Patients should be counselled individually on the safest
50 and the most appropriate method.

# Introduction

Globally family planning is recognised as a life-saving intervention (1). Family planning is an effective method to reduce unintended pregnancies and live birth rates, thus reducing maternal mortality rates. It can also have an influence on education, promotion of gender equality and women’s rights, and can assist in reduction of human immunodeficiency virus (HIV) by use of barrier contraception in the form of male and female condoms (2). This unique intervention could prevent a third of maternal deaths by delaying motherhood, allowing for appropriate interval spacing between births, limiting unplanned and unwanted pregnancies and subsequent abortions. For these reasons the World Health Organization (WHO) issued a report in 2019 recommending family planning as one of the critical strategies in reducing maternal mortality (2,3). Another recommendation is to space birth intervals ~~of~~ by at least 24 months after childbirth to reduce the risk of adverse maternal and neonatal outcomes (4). Evidence has shown that spacing live births by contraception can save more than two million newborns annually (2).

The immediate postpartum period is a unique opportunity to motivate and educate women on family planning options and provide a suitable method for each patient, individualizing her options to best suit her needs and fertility desires (5). There are few studies that describe family planning prevalence in the postpartum period, and even less in South Africa (6,7). The rate of unintended pregnancies is estimated to be as high as 50% in Sub-Saharan Africa (8).

This study aimed to determine the prevalence of family planning use in our setting. By determining the prevalence of uptake of family planning in the postpartum period we can assess if our service delivery is in keeping with the WHO, and Royal College of Obstetrics and Gynaecology (RCOG) recommendations for all reproductive women to receive family planning, as a strategy to prevent unwanted pregnancies, abortions and allow fewer for appropriate interval spacing between births. If these issues are addressed, we can reduce maternal and neonatal morbidity and mortality (1,3–5,9,10). Secondary objectives included assessing the frequency of the different methods of contraception, determining the safety of the methods received by using the WHO medical eligibility category (MEC) scoring system (Table 1), to describe risk factors and co-morbidities of our population and to describe the spacing trends between previous and index pregnancy (11).

**Methods**

# Study Population and Sample

We conducted a cross-sectional, quantitative study at Tygerberg Academic Hospital, a tertiary referral centre in Cape Town, South Africa. A prospective data collection took place from January 2023 till July 2023, until a sample size of 1066 participants was met. The Hospital is a public, academic institution that serves a predominantly low socioeconomic status (SES) and high-risk obstetric population, of majority

African and mixed racial groups. Ethical approval was obtained from the Health Research Ethics Committee (HREC) of Stellenbosch University (S22/09/188), as well as the Western Cape Government for access to records and patient files. Informed consent was waivered, as only medical records were used for data collection with no patient interaction, this waiver was supported and approved by the

HREC.

Participants aged older than 18 years and who were managed in the postpartum period at Tygerberg Academic Hospital were included into the study, regardless of whether the pregnancy ended in a live birth, a stillbirth, or an intrapartum death. Postpartum patients who required longer hospital admission and transfer to the gynecology wards were excluded from the study.

Participants were identified by their discharge records and medical records at the medical clerks in the postnatal wards, on the day of discharge which is usually two- or three-days post-delivery. Maternity records completed by health care professionals during their admission, together with their completed discharge forms were used for data collection and capture. Outstanding laboratory results were searched for on the online laboratory system and follow-up appointments were checked using the hospital`s patient electronic record system. Data were anonymised to ensure privacy and confidentiality of participants’ personal information, and each participant was assigned a unique identifier.

# Measures

To determine the prevalence of family planning in the postpartum period, data was collected on whether the patient had received the family planning method of their choice before discharge. This is marked on the discharge summary as well as the prescription chart. Other information recorded included which method was received, ~~the patients risk factors~~,(specifics please) baseline characteristics (including age, gravidity, parity, booking biochemistry), pregnancy complications, birth outcomes (live birth or stillbirth) and method of feeding (breastfeeding or formula feeding). Other measures investigated was the WHO Medical eligibility criteria score (table 1) and the interval spacing between live births. Information bias was limited by the capturing of medical notes done by the same two research assistants to keep collection and capturing methods consistent.

# Table 1: WHO Medical eligibility criteria (11)

| **Category** | **Definition** | **With clinical**  **Judgement** | **With limited clinical**  **judgement** |
| --- | --- | --- | --- |
| 1. | A condition for which there is no restriction for the use of the contraceptive method. | Use method in any circumstance. | Yes, use the method. |
| 2. | A condition where the advantages of using the method generally outweigh the theoretical or prove risks. | Generally, use method. |  |
| 3. | A condition where the theoretical or proven risks usually outweigh the | Use of the method not usually recommended unless other more appropriate methods | No, do not use the method. |
|  | advantages of using the method. | are not available or not acceptable. |  |
| 4. | A condition that represents an unacceptable health risk if the contraceptive method is used. | Method not to be used. |  |

# Statistical analysis

For the primary outcome of prevalence of family planning in the postpartum period, we calculated a sample size of 1066 anticipating a frequency of 50%, using a 95% ~~confident~~ confidence interval (95% CI) using OpenEpi sample size calculator (12). The 50% frequency was chosen as the most conservative estimate maximising the sample size. Stata version 17.0 was used for the data analysis (StataCorp, Texas 2021). For assessing the prevalence of family planning in postpartum women, we calculated frequency and percentages, with a 95% confident interval (95% CI). Demographics of participants are described using frequency tables and summarized using mean, standard deviation (SD), and range for continuous variables if normally distributed, and median and interquartile ranges for non-normally distributed data. For the secondary objectives, we used frequency tables and graphs, and 95% confident intervals, to summarise data. A bivariant and multivariant secondary analysis was conducted to assess associations between variables and choice of family planning methods.

# Results

Over a period of seven months 1066 participants were recruited for this study and had a recorded family planning method of their choice. The prevalence of family planning in postpartum patients in our setting was 97.5% (95% CI 96.3 - 98.3) (Fig

1).

The maternal characteristics are described in Table 2. The proportion of patients found to be syphilis negative was 96.5%. Of the 196 (18.4%) patients who were HIV positive, the majority (85.7%) had viral loads (VL) of less than 100 copies, indicating viral suppression. In terms of exposure to illegal drugs and alcohol during pregnancy 16.7% of patients admitted to smoking, 1.5% to using substances and 12.1% to the use of alcohol.

The most common risk factors amongst the sample population were advanced maternal age (26.2%), a high body mass index (BMI) above 30kg/m^2^ (58%), chronic hypertension (10.9%), and diabetes mellitus type two (3.5%).

# Table 2: Maternal Baseline Characteristics of postpartum participants at

**Tygerberg Academic Hospital between January and July 2023.**

| **Baseline characteristics (n)** |  |
| --- | --- |
| Age (years) | 30 (25 – 35) |
| Gravidity | 3 (2 – 4) |
| Parity | 2 (2 – 3) |
| Body Mass Index (kg/m^2^) | 32 (26 – 40) |
| **Maternal booking characteristics** | **n (%)** |
| Syphilis (RPR) positive | 39 (3.5) |
| HIV + | 196 (18.4) |
| VL < 100 copies | 168 (85.8) |
| VL > 100 copies | 28 (2.6) |
| Smoking | 176 (16.7) |
| Substance use | 16 (1.5) |
| Alcohol use | 127 (12.1) |
| **Risk factors (n)** | n (%) |
| Advanced maternal age | 279 (26.2) |
| BMI > 30kg/m^2^ | 594 (58.6) |
| BMI > 40kg/m^2^ | 281 (27.7) |
| Chronic hypertension (cHPT) | 116 (10.9) |
| Diabetes mellitus type 2 | 37 (3.5) |

The delivery characteristics are described in Table 3. The proportion of participants who had delivered via caesarean section was 49.3% compared to 50.7% who had undergone a normal vaginal delivery (NVD). All the women whose pregnancy resulted in a live birth (1036) were recorded as breastfeeding their neonate at the time of discharge (Fig 1).

# Table 3: Delivery Characteristics of participants at Tygerberg Academic

**Hospital between January and July 2023.**

| **Mode of delivery** | **n (%)** |
| --- | --- |
| Normal Vaginal Delivery | 540 (50.7) |
| Caesarean Section | 526 (49.3) |
| **Birth Outcomes** | **n (%)** |
| Live Birth | 1036 (97.2) |
| Intrauterine Death | 30 (2.8) |
| **Estimated Gestational Age at delivery**  **(weeks)** | 38 (37-40) |

Majority of the patients requested the progesterone only injectable depo medroxyprogesterone acetate (DMPA) (48.8%). The remainder received the progesterone implant (23.7%) and sterilization by means of tubal ligation (18.4%) and a minority of patients requested the combined oral contraceptives (COC) (2.9%), condoms (0.1%) or the copper intrauterine device (IUCD) (3.6%). Despite receiving individual counselling, a minority (2.5%) of patients declined receiving family planning on discharge (Fig 1).

Using the WHO MEC guidance on safe family planning prescription, each patient was analysed according to their individual risk factors and placed into one of the four categories (Table 1). Of the 1039 patients who had received family planning 47.8% of patients were classified into a MEC category three, 24.2% in MEC category one,

22.5% into MEC category two and 3% into MEC category four (Fig 1). We found that

66% of the patients had spaced their pregnancies more than two years apart (Fig 1).

Table 4 presents the unadjusted (crude) associations between various factors and the choice of family planning methods comparing DMPA to the composite other methods. Women using DMPA are younger on average (29.4 years) compared to those using other methods (30.9 years, OR 0.962, p < 0.001). Women who chose DMPA have a slightly lower BMI than those using other methods (OR 0.983, p = 0.012). HIV-positive women are less likely to use DMPA (OR = 0.651, p = 0.008). Higher parity is associated with a lower likelihood of using Depo (OR = 0.721, p < 0.001), suggesting that women with more children prefer other family planning methods.

**Table 4: Bivariate Analysis to determine associations between DMPA and other methods to maternal variables.**

| **Variable** | **Family Plan Methods** | | **Odds**  **Ratio** | **P-**  **Value** | **95% CI** |
| --- | --- | --- | --- | --- | --- |
|  | **DMPA** | **Other** |  |  |  |
| Age | 29.4 (6.4) | 30.9 (6.1) | 0.962 | <0.001 | (0.94, 0.98) |
| Parity | 29.4 (6.4) | 30.9 (6.1) | 0.721 | <0.001 | (0.652, 0.797) |
| BMI | 32.3(9.3) | 33.8(9.3) | 0.983 | 0.012 | (0.98, 0.99) |
| **HIV Status** |  |  |  |  |  |
| Positive | 80 (15.4%) | 113(21.8%) | 0.651 | 0.008 | (0.475, 0.894) |
| Negative | 440 (84.62%) | 405(78.19%) | 1 |  |  |
| **CHPT** |  |  |  |  |  |
| Yes | 51 (9.82%) | 57 (10.98%) | 0.881 | 0.535 | (0.591, 1.314) |
| No | 469 (90.19%) | 462 (89.02%) | 1 |  |  |

Table 5 shows the adjusted associations after controlling for other factors. Age and HIV status are no longer significant factors for choosing DMPA over other methods. Parity (OR 0.689, p < 0.001) remains statistically significant, indicating that even after adjustment, higher parity decreases the likelihood of choosing DMPA. BMI is a borderline significant (OR 0.986, p = 0.053), suggesting that BMI may still play a role in DMPA use.

**Table 5: Multivariate Analysis describing adjusted associations after controlling for other factors, between variables and DMPA compared to other family planning choices.**

| **Variable** | **Odds Ratio** | **95% CI** | **P-Value** |
| --- | --- | --- | --- |
| Age | 1.007 | (0.980 - 1.033) | 0.626 |
| HIV Status (Positive) | 0.784 | (0.558 - 1.101) | 0.161 |
| Parity | 0.689 | (0.602 - 0.784) | < 0.001 |
| BMI | 0.986 | (0.97 - 1.00) | 0.053 |

# Discussion

Our study found a 97.5% prevalence of family planning in the immediate postpartum period of high-risk obstetrics patients in a low SES. This finding indicates a much higher prevalence rate than other studies reported in South Africa. A study conducted in the rural area of Mpumalanga, South Africa, 2021 by Mandell et al. showed a prevalence of 70.9% family planning use in postpartum women (6) . Whereas prevalence of family planning at a hospital in an urban area in Gauteng Province, South Africa was estimated at 55.3% (7). This high prevalence of uptake of family planning, is aligned with the recommendations of the WHO and RCOG which advocate for all postpartum women to receive family planning as a strategy to reduce maternity and neonatal morbidity and mortality rates, prevent unwanted pregnancies and abortions, and allow adequate interval spacing between births to reduce complications in the next pregnancy (5,9).

The high contraceptive uptake observed in tertiary healthcare settings in South Africa can be attributed to several interconnected factors. Firstly, tertiary facilities often ensure comprehensive access to modern contraceptive methods, including

injectables, implants, and intrauterine devices, addressing the availability challenges common in primary care and rural settings (13). In these facilities, patients benefit from detailed contraceptive counseling and education, fostering informed choices and higher utilization rates (14). Additionally, healthcare providers in tertiary settings are frequently trained specialists in reproductive health, offering expert guidance and support (13). Socioeconomic variables further contribute, as individuals accessing tertiary care may exhibit higher levels of education and autonomy, both of which are positively correlated with contraceptive use (15). The enabling legal framework in South Africa, supporting free contraceptive services for individuals over the age of 12, is better implemented in these advanced facilities (14) Furthermore, cultural and individual empowerment within tertiary settings ensures that women can make autonomous decisions about their reproductive health, unlike other settings where societal norms may pose constraints(15). These factors collectively distinguish tertiary settings in South Africa, where contraceptive uptake is notably higher than in global and local studies, where barriers such as limited education, stigma, and restricted access remain prevalent.

Historical and structural issues have significantly influenced contraceptive uptake patterns in South Africa. During the apartheid era, family planning programs were often tied to population control policies, which disproportionately targeted Black communities. This created mistrust in contraceptive services, as they were perceived as tools of oppression rather than empowerment. Additionally, limited access to healthcare infrastructure in rural areas, coupled with cultural and traditional beliefs about fertility, further shaped contraceptive use (16).

Even in the post-apartheid era, socioeconomic disparities and gender dynamics continue to play a role. Women in low-income communities often face barriers such as inadequate access to healthcare facilities, lack of education about contraceptive options, and stigma surrounding their use. These factors, combined with the legacy of historical injustices, contribute to the complex landscape of contraceptive uptake in South Africa (17).

The proportions of the different family planning methods requested are in keeping with the rest of Sub-Saharan Africa. The DMPA was favored with 48.8% of patients requesting it in our study. In a 2019 systematic review of postpartum contraception use in low- and middle-income countries which included 24 studies, the overall crude pooled estimate of modern contraception use in postpartum women was found to be

41.2%. The most used method was the injectable DMPA, followed by oral contraception and condoms (18). The DMPA has higher failure rates and requires more frequent clinical visits.

Only 3.6% of our participants opted for the copper IUCD, which is considered as a first-line method by many organisations globally (19,20). The Saving Mothers Report in South Africa recommends family planning as part of the national strategy to reduce maternal mortality rates, ~~with~~ specifically aiming to improve postpartum ~~longacting reversible~~ long-acting reversible contraception options and improved accessibility to IUCD and the Implanon (21). These methods are highly effective and do not need to be taken daily, which leads to improved adherence rates.

The WHO developed the medical eligibility criteria as a guideline for safe contraceptive use. The Majority of our patients received a method that would represent a category three (47.8%) or category four (3%) risk. The use of the combined oral contraception method in the immediate postpartum period is classified as a category 4 risk, as it poses an unacceptable health risk. Using the COC in the immediate postpartum period increases the risk of clot formation, such as a pulmonary embolism and myocardial infarcts, which can be life threatening (11).

The major contributing factor for most patients to be assigned a MEC category three risk was the patients who received the injectable DMPA. DMPA has been associated to negatively impact breast milk production and thus impact breast feeding (11). For this reasoning DMPA has been assigned as a category three in the immediate postpartum period. There is also a theoretical risk that high dose progesterone exposure may impact the development of the infant brain. This has been seen in animal studies, but it is unclear if the same impact is seen in humans (11). The quality of evidence both these statements are categorized as low certainty and low grade, which highlights that there is not a direct cause-effect linkage clearly made between these concerns and the use of DMPA. The guideline goes on to acknowledge that in high maternal morbidity and mortality settings with limited access to family planning services, such as low - and lower SES countries, the

DMPA may be one of the only methods accessible to postpartum women and thus may be used (9,11). A systematic review of ~~progesterone only~~ progesterone-onlycontraceptive use among breastfeeding women, conducted a year after the release of the WHO MEC document concluded that there is insufficient evidence to demonstrate adverse breastfeeding outcomes or negative health outcomes infants. This included restricted growth, health-related diseases and impaired development (22).

Interval spacing between pregnancies has been advocated by many organisations worldwide including the WHO as a strategy to lower maternal mortality rate. Majority of our patients had spaced their birth intervals by more than two years, which is in keeping with the recommendation by the WHO of spacing pregnancies more than 24 months apart (4). Providing adequate family planning has been proven to prolong this period (23). The unmet need of family planning in Sub-Saharan Africa has led to reports of inadequate spacing by up to 15% (2).

The bivariant and multivariant analysis gave insight into predictive variables for the choice of DMPA over other family planning options. Parity remains the strongest predictor of Depo use. The OR (0.689, p < 0.001) remains statistically significant, indicating that even after adjustment, higher parity decreases the likelihood of choosing Depo. Women with lower parity often opt for DMPA as a family planning method due to its convenience, effectiveness, and suitability for their reproductive goals. Studies indicate that injectable contraceptives like DMPA are preferred among women who seek long-term, reversible contraception without daily adherence (24). A lower parity may indicate that there is a desire for future fertility and this may lead to choosing a reversible method that could easily be stopped when they wish to complete their families.

The bivariant analysis indicated that BMI, HIV status and age were predictors for determining the likelihood of choosing DMPA over other family planning methods.

Chronic hypertension (OR 0.881, p = 0.535) and late onset pre-eclampsia (OR

1.200, p = 0.216) was found to have no significant association.

In the multivariant analysis age (OR 1.007, p = 0.626) and HIV status (OR 0.784, p = 0.161) lose their significance in the adjusted model, suggesting that their initial associations were likely confounded by other factors. In the adjusted model BMI (OR 0.986, p = 0.053) was found to have a weak association, indicating it could be a possible influence, with a slight reduction in DMPA use as BMI increases.

There could be possible limitations to our results in terms of information bias as the findings of the study are based on data collected from the maternity records and medical notes, which could have missing or incorrectly recoded information. The information collected was done at discharge, however we do not know if these women continued their family planning method or breastfeeding after discharge, as their follow up appointments were not included in this study. The addition of data collection at follow-up appointment’s postpartum would have strengthen this study results and given us insight into possible effects that DPMA might have on breastfeeding, as well as if participants continued with their family planning method long term. This however was no possible for this study patients do not follow up at Tygerberg Academic Hospital, but rather at local clinics dispersed throughout the province which would lead to high rates of fallout from this study.

# Conclusion

The exceptionally high prevalence of uptake of family planning indicates it can be successfully implemented in the postpartum period. Parity appears to be the strongest predictor of choosing DMPA over other family planning methods. Patients should be counselled on an individual basis regarding the safest and most appropriate method to ensure longed-term use and minimise adverse outcomes. Further education and counselling need to be done to encourage women to use long-acting reversible contraception, as recommended by the WHO. Breastfeeding should be encouraged, and family planning methods should be prescribed in a manner to support continued breastfeeding.

# Author contributions

Denise Sproul performed the conceptualization, methodology, formal analysis, investigation, and write up of this manuscript. Gerhard Hanekom provided supervision, reviewing and editing. Michael Mccaul assisted with the formal analysis, supervision, reviewing and editing. We would like to thank Dorothea Van Der Berg and Tamsyn Hall, from the department of obstetrics and gynaecology at Tygerberg Hospital, for data collection and capturing. All the authors are thankful to all the women who contributed to the study.

**Conflicts of interest**

The authors have declared that no competing interests exist.

# References

1. World Health Organization. Programming strategies for Postpartum Family

Planning [Internet]. 2013 [cited 2023 Oct 19]. Available from:

https://www.who.int/publications/i/item/9789241506496

2. Chola L, McGee S, Tugendhaft A, Buchmann E, Hofman K. Scaling up family planning to reduce maternal and child mortality: The potential costs and benefits of modern contraceptive use in South Africa. PLoS One. 2015 Jun 15;10(6).
3. WHO, UNICEF, UNFPA, World Bank Group, United Nations Population Division. Trends in maternal mortality 2000 to 2017. [Internet]. World Health

Organization; 2019 [cited 2023 Oct 19]. Available from: https://www.who.int/publications/i/item/9789241516488

4. Marston C. Report of a WHO Technical Consultation on Birth Spacing

Department of Making Pregnancy Safer (MPS). 2005.

5. Royal College of Obstetrics and Gynaecology. Leading safe choices [Internet].
   2022 [cited 2024 Sep 5]. Available from: https://www.rcog.org.uk/leadingsafechoices
6. Mandell L, Rodriguez VJ, Jones DL. Safer Conception and Family Planning Knowledge, Attitudes, and Practices Among Postpartum Women with HIV in

Rural South Africa. Open Access J Contracept. 2021 Feb;Volume 12:17–25.

7. Olowa SN, Govender I, Saidiya C. The determinants of contraception use amongst female patients attending odi district hospital, Gauteng province,

South Africa. South African Family Practice. 2020;62(1):1–8.

8. Towriss CA, Phillips TK, Brittain K, Zerbe A, Abrams EJ, Myer L. The injection or the injection? Restricted contraceptive choices among women living with HIV. Sex Reprod Health Matters. 2019 Jan 1;27(1):215–27.
9. World Health Organization. World Health Statistics: Monitoring Health for the

Sustained Development Goals [Internet]. World Health Organization ; 2022.

Available from: http://apps.who.int/bookorders.

10. Say L, Chou D, Gemmill A, Tunçalp Ö, Moller AB, Daniels J, et al. Global causes of maternal death: A WHO systematic analysis. Lancet Glob Health.

2014;2(6).

11. World Health Organization. Reproductive Health and Research, World Health Organization. Medical eligibility criteria for contraceptive use. [Internet]. [cited
    2023 Oct 19]. p. 268. Available from: https://www.who.int/publications/i/item/9789241549158
12. Open Epi Sample Size Calculator [Internet]. 2022 [cited 2023 Oct 19].

Available from: https://www.openepi.com

13. Sinegugu. Awareness Factors Affecting Contraceptive Uptake in women attending the Gynaecology Out-Patients Department, at a Tertiary Hospital.

2023.

14. Kriel Y, Milford C, Cordero JP, Suleman F, Steyn PS, Smit JA. A continuum of individual-level factors that influence modern contraceptive uptake and use: perspectives from community members and healthcare providers in Durban,

South Africa. Contracept Reprod Med. 2023 Oct 3;8(1).

15. Sulemana ZS, Gqunu S, Abobo FDN, Halm HA, Obour-Awuku N, Kumi RO, et al. Knowledge and utilisation of family planning services among tertiary students in Northern Ghana: The case of College of Nursing and Midwifery, Nalerigu. Afr J Reprod Health. 2024 Jul 9;28(5):55–66.
16. Tshiki N. A South African history of contraceptive use: The Eastern Cape

Experience, 1960-1980. 2024.

17. Kriel Y, Milford C, Cordero JP, Suleman F, Steyn PS, Smit JA. A continuum of individual-level factors that influence modern contraceptive uptake and use: perspectives from community members and healthcare providers in Durban, South Africa. Contracept Reprod Med. 2023 Oct 3;8(1).
18. Dev R, Kohler P, Feder M, Unger JA, Woods NF, Drake AL. A systematic review and meta-analysis of postpartum contraceptive use among women in low- And middle-income countries. Vol. 16, Reproductive Health. BioMed Central Ltd.; 2019.
19. Kamuyango A, Yu T, Ao CK, Hu SC, Hou WH, Tseng CC, et al. The publicsector family planning program impact scores in association with long-acting reversible contraceptive use among young women in 22 Sub-Saharan African countries: A pooled multi-level analysis. Contraception. 2022 Apr 1;108:44–9.
20. Chersich MF, Wabiri N, Risher K, Shisana O, Celentano D, Rehle T, et al. Contraception coverage and methods used among women in South Africa: A national household survey. South African Medical Journal. 2017 Apr 1;107(4):307–14.
21. Moodley j, Fawcus S, Pattinson R. Saving Mothers and Babies 2017-2019: Executive Summary Final report the effect of the first wave of Covid-19 on use

of maternal and reproductive health services and maternal and perinatal deaths in South Africa. 2020.

22. Phillips SJ, Tepper NK, Kapp N, Nanda K, Temmerman M, Curtis KM. Progestogen-only contraceptive use among breastfeeding women: a systematic review. Vol. 94, Contraception. Elsevier USA; 2016. p. 226–52.
23. Karra M, Maggio D, Guo M, Ngwira B, Canning D. The causal effect of a family planning intervention on women’s contraceptive use and birth spacing. 2022;
24. Mwafulirwa B. Women’s experiences of amenorrhea following Depo-Provera use at a district hospital in Malawi. ACADEMIA. 2016;

Figure	[Click here to access/download;Figure;Figure 1.jpeg](https://www2.cloud.editorialmanager.com/pone/download.aspx?id=40021648&guid=993c48a9-79fb-44cf-a3d4-0c879312dbec&scheme=1)

[Click here to access/download](https://www2.cloud.editorialmanager.com/pone/download.aspx?id=39898568&guid=03449479-49f3-47a4-9403-c9a386f3551f&scheme=1)

[Supporting Information](https://www2.cloud.editorialmanager.com/pone/download.aspx?id=39898568&guid=03449479-49f3-47a4-9403-c9a386f3551f&scheme=1)

[Bivariate_and_Multivariate_Analysis (002).docx](https://www2.cloud.editorialmanager.com/pone/download.aspx?id=39898568&guid=03449479-49f3-47a4-9403-c9a386f3551f&scheme=1)

[Click here to access/download](https://www2.cloud.editorialmanager.com/pone/download.aspx?id=39898569&guid=15ca1286-ef00-4cd2-a267-c0b316efda72&scheme=1)

[Supporting Information](https://www2.cloud.editorialmanager.com/pone/download.aspx?id=39898569&guid=15ca1286-ef00-4cd2-a267-c0b316efda72&scheme=1)

[Interpretation of the Tables_Denise (002).docx](https://www2.cloud.editorialmanager.com/pone/download.aspx?id=39898569&guid=15ca1286-ef00-4cd2-a267-c0b316efda72&scheme=1)

[Click here to access/download](https://www2.cloud.editorialmanager.com/pone/download.aspx?id=39985649&guid=315d0fc5-f424-41fd-bc77-4a3434ff7058&scheme=1)

[Supporting Information](https://www2.cloud.editorialmanager.com/pone/download.aspx?id=39985649&guid=315d0fc5-f424-41fd-bc77-4a3434ff7058&scheme=1)

[Data - Anonymized.xlsx](https://www2.cloud.editorialmanager.com/pone/download.aspx?id=39985649&guid=315d0fc5-f424-41fd-bc77-4a3434ff7058&scheme=1)

Revised Manuscript with Track Changes

**Prevalence and sociodemographic risk and morbidities associated**

1

**with contraceptive uptake among postpartum women at**

**Tygerberg**

2

**Academic Hospital**

**, Cape Town:**

**A cross**

**-**

**sectional study**

**.**

3

Denise Sproul

1

,

2

, Michael M

cC

aul

1

, Gerhard Hanekom

2

.

4

5

1

Divison of Epidemiology and Biostatistics, Department of Global Health,

6

Stellenbosch University, Cape Town, South Africa.

7

2

Department of Obstetrics and Gynaecology, Stellenbosch University, Cape Town,

8

South Africa.

9

10

**Corresponding author:**

11

Denise Sproul

.

12

Department of Obstetrics and Gynaecology

,

Stellenbosch University

,

13

Francie Van Zyl Drive

,

Cape Town, South Africa.

14

Email:

dsproul@sun.ac.za

15

**ORCHID iD:**

16

Denise Sproul:

[9](https://orcid.org/0009-0003-2138-0495)

[000](https://orcid.org/0009-0003-2138-0495)

[-](https://orcid.org/0009-0003-2138-0495)

[3](https://orcid.org/0009-0003-2138-0495)

[000](https://orcid.org/0009-0003-2138-0495)

[-](https://orcid.org/0009-0003-2138-0495)

[8](https://orcid.org/0009-0003-2138-0495)

[213](https://orcid.org/0009-0003-2138-0495)

[-](https://orcid.org/0009-0003-2138-0495)

[049](https://orcid.org/0009-0003-2138-0495)

[5](https://orcid.org/0009-0003-2138-0495)

17

Gerhard Hanekom: 0000

-

0002

-

2832

-

9436

18

Michael McCaul:

[0](https://orcid.org/0000-0002-2730-6478)

[000](https://orcid.org/0000-0002-2730-6478)

[-](https://orcid.org/0000-0002-2730-6478)

[000](https://orcid.org/0000-0002-2730-6478)

[2](https://orcid.org/0000-0002-2730-6478)

[-](https://orcid.org/0000-0002-2730-6478)

[0](https://orcid.org/0000-0002-2730-6478)

[273](https://orcid.org/0000-0002-2730-6478)

[-](https://orcid.org/0000-0002-2730-6478)

[8](https://orcid.org/0000-0002-2730-6478)

[647](https://orcid.org/0000-0002-2730-6478)

19

20

**Short Title:**

21

**High prevalence of postpartum family planning found in South Africa.**

22

23

**Commented [DS1]:**

Title edi

ted as requested

**Commented [DS2]:**

Academic qualifications removed

as requested

**Commented [DS3]:**

ORCHID iD numbers added as

requested

25

26

27

28

29

30

31

32

33

34

35

36

**Abstract**

24

The

immediate postpartum period is a unique opportunity to motivate and educate

women on family planning options

.

This study aimed to determine the prevalence of

family planning use in

our

setting.

Secondary objectives include determining the

frequency of the

various methods used, safety of the method, common risk factors,

the interval spacing between births

and associations

with family planning method

choices

.

A

cross

-

sectional

study

was conducted

at Tygerberg

Academic

Hospital,

South Africa

,

which serves a p

redominantly low socioeconomic

status

and high

-

risk

obstetric population.

Medical records were assessed

prospectively

from January

2023

till July

2023

. Data was collected and captured from maternity medical records

on the day of discharge. The statistical analysis was done using frequencies and

percentages, with 95% confidence intervals (95%CI).

A secondary bivariant analysis

was done to evaluate the as

sociations between these variables.

Of the 1066

participants included, 97.5% (95%CI 96.5

-

received family planning. Majority of

98.5)

the patients (48.8%) requested the injectable depo medroxyprogesterone acetate.

These

patients who chose this meth

o

d were

o

n average

younger (

OR

0.962

,

p<0.001

)

, had a lower BMI

(

OR

0.983

, p

=0.012)

,

and

are a

higher parity

(

OR 0.721,

p<0.001)

. HIV positive women were also

% less likely (OR 0.651, p= 0.008)

35

to

choose this method compared to other methods.

Of the 1039

patients who had

received family planning

,

47.8

% were classified into a

World Health Organization

medical eligibility category

three.

This was almost exclusively attributed due to

patients who were breastfeeding and requested the depo medroxyprogesterone

acetate as their family planning method postpartum.

Majority (66%) of the patients

had spaced their pregnancies more than two years ap

art.

The exceptionally high

prevalence of uptake of family planning indicates it

can be successfully

implemented

**Commented [DS4]:**

Added statistical results as

requested

37

38

39

40

41

42

43

44

45

46

47

48

49 in the postpartum period. Patients should be counselled individually on the safest
50 and most appropriate method.

# Introduction

Globally family planning is recognised as a life-saving intervention (1). Family planning is an effective method to reduce unintended pregnancies and live birth rates, thus reducing maternal mortality rates. It can also have an influence on education, promotion of gender equality and women’s rights, and can assist in reduction of human immunodeficiency virus (HIV) by use of barrier contraception in the form of male and female condoms (2). This unique intervention could prevent a third of maternal deaths by delaying motherhood, allowing for appropriate interval spacing between births, limiting unplanned and unwanted pregnancies and subsequent abortions. For these reasons the World Health Organization (WHO) issued a report in 2019 recommending family planning as one of the critical strategies in reducing maternal mortality (2,3). Another recommendation is to space birth intervals of at least 24 months after childbirth to reduce the risk of adverse maternal and neonatal outcomes (4). Evidence has shown that spacing of live births by contraception can save more than two million newborns annually (2).

The immediate postpartum period is a unique opportunity to motivate and educate women on family planning options and provide a suitable method for each patient, individualizing her options to best suit her needs and fertility desires (5). There are few studies that describe family planning prevalence in the postpartum period, and even less in South Africa (6,7). The rate of unintended pregnancies is estimated to be as high as 50% in Sub-Saharan Africa (8).

74

75

76

77

78

79

80

81

82

83

84

85

86

This study aimed to determine the prevalence of family planning use in our setting.

By

determining the prevalence of uptake of family planning in the postpartum period we

can assess if our service delivery is in keeping with the WHO, and Royal College of

Obstetrics and Gynaecology (RCOG) recommendations for all reproductive women to

rece

ive family planning, as a strategy to prevent unwanted pregnancies, abortions and

allow for appropriate interval spacing between births. If these issues are addressed,

we can reduce maternal and neonatal morbidity and mortality

3

(1

,

–

5

,

9,10)

.

Secondary

objectives included

assessing the frequency of the different methods of contraception,

determining the safety of the methods

received

by using the WHO

medical eligibility

category

(

MEC

)

scoring system

Table

1)

(

, to describe risk factors and co

-

mo

rbidities

of our population and to describe the spacing trends between previous and index

pregnancy

(11)

.

**Methods**

87

**Study Population and Sample**

We conducted a cross

-

sectional, quantitative study at Tygerberg

Academic

Hospital,

a tertiary referral centre in Cape Town, South Africa.

A

prospective d

ata

collection

took place from January 2023 till July 2023

,

until

a sample size of 1066 participants

was met

.

The Hospital is a public

,

academic institution that serves a predominantly

low socioeconomic status (SES) and high

-

risk obstetric population

, of majority

African and mixed racial groups

.

Ethical approval was obtained from the Health

Research Ethics Committee

(

HREC

)

of Stellenbosch

University (S22/09/188), as

well as the Western Cape Government for access to records and patient files.

Informed consent was waivered, as

only medical records

were used for data

**Commented [DS5]:**

The red text indicates the

revisions requested for this section

88

89

90

91

92

93

94

95

96

97

collection with no patient interaction, this waiver was supported and approved by the

HREC.

Participants aged older than 18 years and who were managed in the postpartum period at Tygerberg Academic Hospital were included into the study, regardless of whether the pregnancy ended in a live birth, a stillbirth, or an intrapartum death. Postpartum patients who required longer hospital admission and transfer to the gynecology wards were excluded from the study.

Participants were identified by their discharge records and medical records at the medical clerks in the postnatal wards, on the day of discharge which is usually two- or three-days post-delivery. Maternity records completed by health care professionals during their admission, together with their completed discharge forms were used for data collection and capture. Outstanding laboratory results were searched for on the online laboratory system and follow up appointments were checked using the hospitals patient electronic record system. Data were anonymised to ensure privacy and confidentiality of participants’ personal information, and each participant was assigned a unique identifier.

## Measures

To determine the prevalence of family planning in the postpartum period, data was collected on whether the patient had received the family planning method of their choice before discharge. This is marked on the discharge summary as well as the prescription chart. Other information recorded included which method was received, the patients risk factors, baseline characteristics (including age, gravidity, parity,

136

137

138

139

140

141

142

143

144

145

146

size.

Stata version 17.0 was used for the data analysis (StataCorp, Texas 2021). For

assessing prevalence of family planning in postpartum women, we calculated

frequency and percentages, with a 95% confident interval (95% CI).

Demographics

of participants are de

scribed using frequency tables and summarized using mean,

standard deviation (SD), and range for continuous variables if normally distributed

,

and m

edian and

interquartile

ranges for non

-

normally distributed

data.

For the

secondary objectives, we used freq

uency tables and graphs, and 95% confident

intervals, to summarise data.

A

bivariant

and multivariant secondary analysis was

conducted to assess

associations between variables and

choice of family planning

methods.

**Results**

147

Over a period of seven

months 1066 participants were recruited for this study and

had a recorded family planning method of their choice. The prevalence of family

planning in postpartum patients in our setting was 97.5% (95% CI 96.3

-

98.3) (

Fi

g

1)

.

The maternal characteristics

are

described

in Table

2

. The proportion of patients

found to be syphilis negative was 96.5%

.

Of the 196 (18.4%

)

patients who were HIV

positive,

majority

(

%

85.7

)

had viral loads (VL) of less than 100 copies, indicating viral

suppression. In terms of exposure to illegal drugs and alcohol during pregnancy

% of patients admitted to smoking, 1.5% to using substances and 12.1% to the

16.7

use of alcohol.

**Commented [DS7]:**

Statement includes results

representation and analysis for normally and skewed

results.

**Commented [DS8]:**

Additional analysis done as

requested.

**Commented [DS9]:**

Section revised as requested.

Results table split from 1 table into 2 tables to separate

maternal characteristics (table2) and

delivery

characteristics (table 3)

**Commented [DS10]:**

Edited as requested

148

149

150

151

152

153

154

155

156

157

The delivery characteristics are described in Table 3. The proportion of participants who had delivered via caesarean section was 49.3% compared to 50.7% who had undergone a normal vaginal delivery (NVD). All the women whose pregnancy resulted in a live birth (1036) were recorded as breastfeeding their neonate at the time of discharge (Fig 1).

**Table 3: Delivery Characteristics of participants at Tygerberg Academic Hospital between January and July 2023.**

| **Mode of delivery** | **n (%)** |
| --- | --- |
| Normal Vaginal Delivery | 540 (50.7) |
| Caesarean Section | 526 (49.3) |
| **Birth Outcomes** | **n (%)** |
| Live Birth | 1036 (97.2) |
| Intrauterine Death | 30 (2.8) |
| **Estimated Gestational Age at delivery**  **(weeks)** | 38 (37-40) |

Majority of the patients requested the progesterone only injectable depo medroxyprogesterone acetate (DMPA) (48.8%). The remainder received the progesterone implant (23.7%) and sterilization by means of tubal ligation (18.4%) and a minority of patients requested the combined oral contraceptives (COC) (2.9%), condoms (0.1%) or the copper intrauterine device (IUCD) (3.6%). Despite receiving individual counselling, a minority (2.5%) of patients declined receiving family planning on discharge (Fig 1).

Using the WHO MEC guidance on safe family planning prescription, each patient was analysed according to their individual risk factors and placed into one of the four categories (Table 1). Of the 1039 patients who had received family planning 47.8% of patients were classified into a MEC category three, 24.2% in MEC category one,

22.5% into MEC category two and 3% into MEC category four (Fig 1). We found that

66% of the patients had spaced their pregnancies more than two years apart (Fig 1).

Table 4 presents the unadjusted (crude) associations between various factors and the choice of family planning methods comparing DMPA to the composite other methods. Women using DMPA are younger on average (29.4 years) compared to those using other methods (30.9 years, OR 0.962, p < 0.001). Women who chose DMPA have a slightly lower BMI than those using other methods (OR 0.983, p = 0.012). HIV-positive women are less likely to use DMPA (OR = 0.651, p = 0.008). Higher parity is associated with a lower likelihood of using Depo (OR = 0.721, p < 0.001), suggesting that women with more children prefer other family planning methods.

**Table 4: Bivariate Analysis to determine associations between DMPA and other methods to maternal variables.**

| **Variable** | **Family Plan Methods** | | **Odds**  **Ratio** | **P-**  **Value** | **95% CI** |
| --- | --- | --- | --- | --- | --- |
|  | **DMPA** | **Other** |  |  |  |
| Age | 29.4 (6.4) | 30.9 (6.1) | 0.962 | <0.001 | (0.94, 0.98) |
| Parity | 29.4 (6.4) | 30.9 (6.1) | 0.721 | <0.001 | (0.652, 0.797) |
| BMI | 32.3(9.3) | 33.8(9.3) | 0.983 | 0.012 | (0.98, 0.99) |
| **HIV Status** |  |  |  |  |  |
| Positive | 80 (15.4%) | 113(21.8%) | 0.651 | 0.008 | (0.475, 0.894) |
| Negative | 440 (84.62%) | 405(78.19%) | 1 |  |  |
| **CHPT** |  |  |  |  |  |
| Yes | 51 (9.82%) | 57 (10.98%) | 0.881 | 0.535 | (0.591, 1.314) |
| No | 469 (90.19%) | 462 (89.02%) | 1 |  |  |

Table 5 shows the adjusted associations after controlling for other factors. Age and HIV status are no longer significant factors for choosing DMPA over other methods. Parity (OR 0.689, p < 0.001) remains statistically significant, indicating that even after adjustment, higher parity decreases the likelihood of choosing DMPA. BMI is a borderline significant (OR 0.986, p = 0.053), suggesting that BMI may still play a role in DMPA use.

**Table 5: Multivariate Analysis describing adjusted associations after controlling for other factors, between variables and DMPA compared to other family planning choices.**

| **Variable** | **Odds Ratio** | **95% CI** | **P-Value** |
| --- | --- | --- | --- |
| Age | 1.007 | (0.980 - 1.033) | 0.626 |
| HIV Status (Positive) | 0.784 | (0.558 - 1.101) | 0.161 |
| Parity | 0.689 | (0.602 - 0.784) | < 0.001 |
| BMI | 0.986 | (0.97 - 1.00) | 0.053 |

209

210

211

212

213

214

215

216

217

218

219

220

**Discussion**

208

Our study found a 97.5% prevalence of family planning in the immediate postpartum

period of high

-

risk obstetrics patients in a low SES. This finding indicate

s

a much

higher prevalence rate than other studies reported in South Africa.

A

study conducted

in

the

rural area of Mpumalanga, South Africa

,

2021

by Mandell et al. showed a

prevalence of 70.9% family planning use in postpartum women

(6)

.

Whereas

prevalence of family planning at a hospital in an urban area in Gauteng Province,

South Africa was estimated at 55.3%

(7)

.

This high prevalence of uptake of family

planning, is aligned with the recommendations of the WHO and RCOG which advocate

for all postpartum women to receive family planning as a strategy to reduce maternity

and neonatal morbidity and mortality rates, prevent unwanted pregnancies and

abortions, and allow adequate interval spacing between births to reduce complications

in the

next pregnancy

(5

,

9)

.

The high contraceptive uptake observed in tertiary healthcare settings in South Africa

can be attributed to several interconnected factors. Firstly, tertiary facilities often

ensure comprehensive access to modern contraceptive met

hods, including

injectables, implants, and intrauterine devices, addressing the availability challenges

common in primary care and rural settings

(13)

. In these facilities, patients benefit from

detailed contraceptive counseling and education, fostering informed

choices and

higher utilization rates

(14)

.

Additionally, healthcare providers in tertiary settings are

frequently trained specialists in r

eproductive health, offering expert guidance and

support

(13)

.

Socioeconomic variables further contribute, as individuals accessing

tertiary care may exhibit higher levels of education and autonomy, both of which are

positively correlated with contracept

ive use

(15)

. The enabling legal framework in

**Commented [DS12]:**

Added to discussion as

requested rega

rding high uptake rates

221

222

223

224

225

226

227

228

229

230

231

232

South Africa, supporting free contraceptive services for individuals over the age of 12,

233

is better implemented in these advanced facilities

(14)

Furthermore, cultural and

234

individual empowerment within terti

ary settings ensures that women can make

235

autonomous decisions about their reproductive health, unlike other settings where

236

societal norms may pose constraints

(15)

. These factors collectively distinguish tertiary

237

settings in South Africa, where contracept

ive uptake is notably higher than in global

238

and local studies, where barriers such as limited education, stigma, and restricted

239

access remain prevalent.

240

241

H

istorical and structural issues

have significantly influenced contraceptive uptake

242

patterns in

South Africa. During the apartheid era, family planning programs were often

243

tied to population control policies, which disproportionately targeted Black

244

communities. This created mistrust in contraceptive services, as they were perceived

245

as tools of oppres

sion rather than empowerment. Additionally, limited access to

246

healthcare infrastructure in rural areas, coupled with cultural and traditional beliefs

247

about fertility, further shaped contraceptive use

(16)

.

248

249

Even in the post

-

apartheid era, socioeconomic d

isparities and gender dynamics

250

continue to play a role. Women in low

-

income communities often face barriers such

251

as inadequate access to healthcare facilities, lack of education about contraceptive

252

options, and stigma surrounding their use. These factors,

combined with the legacy of

253

historical injustices, contribute to the complex landscape of contraceptive uptake in

254

South Africa

(17)

.

255

256

**Commented [DS13]:**

Social, cultural and historian

factors affecting FP

The proportions of the different family planning methods requested are in keeping with the rest of Sub-Saharan Africa. The DMPA was favored with 48.8% of patients requesting it in our study. In a 2019 systematic review of postpartum contraception use in low- and middle-income countries which included 24 studies, the overall crude pooled estimate of modern contraception use in postpartum women was found to be 41.2%. The most used method was the injectable DMPA, followed by oral contraception and condoms (18). The DMPA has higher failure rates and requires more frequent clinical visits.

Only 3.6% of our participants opted for the copper IUCD, which is considered as a first-line method by many organisations globally (19,20). The Saving Mothers Report in South Africa recommends family planning as part of the national strategy to reduce maternal mortality rates, with specifically aiming to improve postpartum longacting reversable contraception options and improved accessibility to IUCD and the Implanon (21). These methods are highly effective and do not need to be taken daily, which leads to improved adherence rates.

The WHO developed the medical eligibility criteria as a guideline for safe contraceptive use. Majority of our patients received a method that would represent a category three (47.8%) or category four (3%) risk. The use of the combined oral contraception method in the immediate postpartum period is classified as a category 4 risk, as it poses an unacceptable health risk. Using the COC in the immediate postpartum period increases the risk of clot formation, such as a pulmonary embolism and myocardial infarcts, which can be life threatening (11).

The major contributing factor for most patients to be assigned a MEC category three risk was the patients who received the injectable DMPA. DMPA has been associated to negatively impact breast milk production and thus impact breast feeding (11). For this reasoning DMPA has been assigned as a category three in the immediate postpartum period. There is also a theoretical risk that high dose progesterone exposure may impact the development of the infant brain. This has been seen in animal studies, but it is unclear if the same impact is seen in humans (11). The quality of evidence both these statements are categorized as low certainty and low grade, which highlights that there is not a direct cause-effect linkage clearly made between these concerns and the use of DMPA. The guideline goes on to acknowledge that in high maternal morbidity and mortality settings with limited access to family planning services, such as low - and lower SES countries, the DMPA may be one of the only methods accessible to postpartum women and thus may be used (9,11). A systematic review of progesterone only contraceptive use among breastfeeding women, conducted a year after the release of the WHO MEC document concluded that there is insufficient evidence to demonstrate adverse breastfeeding outcome or negative health outcomes infants. This included restricted growth, health related diseases and impaired development (22).

Interval spacing between pregnancies has been advocated by many organisations worldwide including the WHO as a strategy to lower maternal mortality rate. Majority of our patients had spaced their birth intervals by more than two years, which is in keeping with the recommendation by the WHO of spacing pregnancies more than 24 months apart (4). Providing adequate family planning has been proven to prolong this period (23). The unmet need of family planning in Sub-Saharan Africa has led to reports of inadequate spacing by up to 15% (2).

The bivariant and multivariant analysis gave insight into predictive variables for the

choice of DMPA over other family planning options. Parity remains the strongest

predictor of Depo use.

The OR (0.689, p < 0.001) remains statistically significant,

306

indicating that even after adjustment, higher pari

ty decreases the likelihood of

307

choosing Depo.

Women with lower parity often opt for

DMPA

as a family planning

308

method due to its convenience, effectiveness, and suitability for their reproductive

309

goals. Studies indicate that injectable contraceptives like D

MPA

are preferred among

310

women who seek long

-

term, reversible contraception without daily adherence

(24)

.

A

311

lower parity may indicate that there is a desire for future fertility and this may

lead to

312

choosing a reversible method that could easily be sto

pped when they wish to

complete

313

their families.

314

The

bivariant analysis indicated that BMI, HIV status and age

were predictors for

315

determining the likelihood of choosing DMPA over other family planning methods.

316

Chronic hypertension

(

OR 0.881, p =

0.535)

and late onset pre

-

eclampsia

(

OR

317

, p =

0

1.200

.216)

was

found to have no significant association.

318

I

n the multiva

riant analysis age

= 0.6

(

OR 1.007, p

26)

and HIV status

(

OR 0.784, p =

319

0.161)

lose their significance

in the adjusted model, suggesting that the

ir initial

320

associations were likely confounded by other factors. In the adjusted

model BMI

(

OR

321

= 0.053)

, p

0.986

was found to have a weak association, indicating it

could be a

322

possible influence, with a slight reduction in DMPA use as BMI increases.

323

There could be possible

limitations

to our results

in terms of information bias as the

324

findings of the study are based on data collected from the maternity records and

325

medical notes, which could have missing or incorrectly recoded information.

The

326

infor

mation collected was done at discharge, however we do not know if these

327

women continued their family planning method or breastfeeding after discharge, as

328

their follow up appointments were not included in this study.

The addition of data

329

**Commented [DS14]:**

Added discussion points to

include the secondary analysis

**Commented [DS15]:**

Limitations to the study are

discussed in this paragraph

collection at follow-up appointment’s postpartum would have strengthen this study results and given us insight into possible effects that DPMA might have on breastfeeding, as well as if participants continued with their family planning method long term. This however was no possible for this study patients do not follow up at Tygerberg Academic Hospital, but rather at local clinics dispersed throughout the province which would lead to high rates of fallout from this study.

# Conclusion

The exceptionally high prevalence of uptake of family planning indicates it can be successfully implemented in the postpartum period. Parity appears to be the strongest predictor of choosing DMPA over other family planning methods. Patients should be counselled on an individual basis regarding the safest and most appropriate method to ensure long term use and minimise adverse outcomes. Further education and counselling need to be done to encourage women to use long-acting reversible contraception, as recommend by the WHO. Breastfeeding should be encouraged, and family planning methods should be prescribed in a manner to support continued breastfeeding.

# Author contributions

Denise Sproul performed the conceptualization, methodology, formal analysis, investigation, and write up of this manuscript. Gerhard Hanekom provided supervision, reviewing and editing. Michael Mccaul assisted with the formal analysis, supervision, reviewing and editing. We would like to thank Dorothea Van Der Berg

353

354

355

356	357 **~~Funding~~**

358

359

360

361

362

363

and Tamsyn Hall, from the department of obstetrics and gynaecology at Tygerberg

Hospital, for data collection and capturing. All the authors are thankful to all the

women who contributed to the s

tudy.

~~We would like to acknowledge the Temporary Research funding support from~~

~~Stellenbosch University’s Subcommittee C of the Research Committee. As well as~~

~~the funding received from the~~

~~GELA project which is part of the EDCTP2 programme~~

~~supported by the European Union (grant number RI2020S~~

~~-~~

~~3303~~

~~-~~

~~GELA). The views~~

~~and opinions of authors expressed herein do not necessarily state or reflect those of~~

EDCTP.

The funders had no role in study design, data collection and analysis,

decision to pu

blish, or preparation of the manuscript.

**Conflicts of interest**

366

The authors have declared that no competing interests exist.

World Health Organization. Programming strategies for Postpartum Family Planning

[

Internet]. 2013 [cited 2023

Oct 19]. Available from:

https://www.who.int/publications/i/item/9789241506496

Chola L, McGee S, Tugendhaft A, Buchmann E, Hofman K. Scaling up family

planning to reduce maternal and child mortality: The potential costs and benefits of

modern contracept

ive use in South Africa. PLoS One. 2015 Jun 15;10(6).

WHO, UNICEF, UNFPA, World Bank Group, United Nations Population Division.

Trends in maternal mortality 2000 to 2017. [Internet]. World Health Organization; 2019

**Commented [DS16]:**

Removed as requested

364

365

367

368

# 369 References

370	1.

371

372

373	2.

374

375

376	3. 377

[cited 2023 Oct 19]. Available from: https://www.who.int/publications/i/item/9789241516488

4. Marston C. Report of a WHO Technical Consultation on Birth Spacing Department of Making Pregnancy Safer (MPS). 2005.
5. Royal College of Obstetrics and Gynaecology. Leading safe choices [Internet]. 2022

[cited 2024 Sep 5]. Available from: https://www.rcog.org.uk/leadingsafechoices

6. Mandell L, Rodriguez VJ, Jones DL. Safer Conception and Family Planning Knowledge, Attitudes, and Practices Among Postpartum Women with HIV in Rural South Africa. Open Access J Contracept. 2021 Feb;Volume 12:17–25.
7. Olowa SN, Govender I, Saidiya C. The determinants of contraception use amongst female patients attending odi district hospital, Gauteng province, South Africa. South African Family Practice. 2020;62(1):1–8.
8. Towriss CA, Phillips TK, Brittain K, Zerbe A, Abrams EJ, Myer L. The injection or the injection? Restricted contraceptive choices among women living with HIV. Sex Reprod Health Matters. 2019 Jan 1;27(1):215–27.
9. World Health Organization. World Health Statistics: Monitoring Health for the Sustained Development Goals [Internet]. World Health Organization ; 2022. Available from: http://apps.who.int/bookorders.
10. Say L, Chou D, Gemmill A, Tunçalp Ö, Moller AB, Daniels J, et al. Global causes of maternal death: A WHO systematic analysis. Lancet Glob Health. 2014;2(6).
11. World Health Organization. Reproductive Health and Research, World Health Organization. Medical eligibility criteria for contraceptive use. [Internet]. [cited 2023 Oct 19]. p. 268. Available from:

https://www.who.int/publications/i/item/9789241549158

12. Open Epi Sample Size Calculator [Internet]. 2022 [cited 2023 Oct 19]. Available from: https://www.openepi.com
13. Sinegugu. Awareness Factors Affecting Contraceptive Uptake in women attending the Gynaecology Out-Patients Department, at a Tertiary Hospital. 2023.
14. Kriel Y, Milford C, Cordero JP, Suleman F, Steyn PS, Smit JA. A continuum of individual-level factors that influence modern contraceptive uptake and use:

perspectives from community members and healthcare providers in Durban, South Africa. Contracept Reprod Med. 2023 Oct 3;8(1).

15. Sulemana ZS, Gqunu S, Abobo FDN, Halm HA, Obour-Awuku N, Kumi RO, et al. Knowledge and utilisation of family planning services among tertiary students in Northern Ghana: The case of College of Nursing and Midwifery, Nalerigu. Afr J Reprod Health. 2024 Jul 9;28(5):55–66.
16. Tshiki N. A South African history of contraceptive use: The Eastern Cape Experience, 1960-1980. 2024.
17. Kriel Y, Milford C, Cordero JP, Suleman F, Steyn PS, Smit JA. A continuum of individual-level factors that influence modern contraceptive uptake and use:

perspectives from community members and healthcare providers in Durban, South Africa. Contracept Reprod Med. 2023 Oct 3;8(1).

18. Dev R, Kohler P, Feder M, Unger JA, Woods NF, Drake AL. A systematic review and meta-analysis of postpartum contraceptive use among women in low- And middleincome countries. Vol. 16, Reproductive Health. BioMed Central Ltd.; 2019.
19. Kamuyango A, Yu T, Ao CK, Hu SC, Hou WH, Tseng CC, et al. The public-sector family planning program impact scores in association with long-acting reversible contraceptive use among young women in 22 Sub-Saharan African countries: A pooled multi-level analysis. Contraception. 2022 Apr 1;108:44–9.
20. Chersich MF, Wabiri N, Risher K, Shisana O, Celentano D, Rehle T, et al. Contraception coverage and methods used among women in South Africa: A national household survey. South African Medical Journal. 2017 Apr 1;107(4):307–14.
21. Moodley j, Fawcus S, Pattinson R. Saving Mothers and Babies 2017-2019: Executive Summary Final report the effect of the first wave of Covid-19 on use of maternal and reproductive health services and maternal and perinatal deaths in South Africa. 2020.
22. Phillips SJ, Tepper NK, Kapp N, Nanda K, Temmerman M, Curtis KM. Progestogenonly contraceptive use among breastfeeding women: a systematic review. Vol. 94, Contraception. Elsevier USA; 2016. p. 226–52.
23. Karra M, Maggio D, Guo M, Ngwira B, Canning D. The causal effect of a family planning intervention on women’s contraceptive use and birth spacing. 2022;
24. Mwafulirwa B. Women’s experiences of amenorrhea following Depo-Provera use at a district hospital in Malawi. ACADEMIA. 2016;

Response to Reviewers

Dear Editor in Chief,

Thank you for the opportunity to re-submit our article to PLOS ONE journal. Please find below a rebuttal table outlining the changes and revisions requested for our submission.

| Changes requested | Revision action. |
| --- | --- |
| Title: Authors should remove the academic qualifications from the authors' list The title needs review. "A cross-sectional prevalence study demonstrating a high uptake of postpartum family planning in a high risk setting in South Africa." what  "high risk" are the authors referring to? HIV prevalence? poor contraceptive uptake? etc. I suggest: "Prevalence and sociodemographic risk and morbidities associated with contraceptive uptake among postpartum women at ...... A cross-sectional study". This is a suggestion that can be reviewed by the authors. | The title has been revised and edited accordingly. The authors academic qualifications have been removed. |
| Authors should use continuous number line when submitting for efficient review | Continuous number line have been inserted. |
| PLOS requires an ORCID iD for the corresponding author in Editorial Manager on papers submitted after December 6th, 2016. Please ensure that you have an ORCID iD and that it is validated in Editorial Manager. To do this, go to ‘Update my Information’ (in the upper left-hand corner of the main menu), and click on the Fetch/Validate link next to the ORCID field. This will take you to the ORCID site and allow you to create a new iD or authenticate a pre-existing iD in Editorial Manager. | Orchid numbers have been added and updated.  Denise Sproul: [0009-0003-2138-](https://orcid.org/0009-0003-2138-0495)  [0495](https://orcid.org/0009-0003-2138-0495)  Gerhard Hanekom: 0000-0002-  2832-9436  Michael McCaul: [0000-0002-2730-](https://orcid.org/0000-0002-2730-6478)  [6478](https://orcid.org/0000-0002-2730-6478) |
| Thank you for stating the following financial disclosure: We would like to acknowledge the Temporary Research funding support from  Stellenbosch University’s Subcommittee C of the Research Committee. As well as the funding received from the GELA project which is part of the | All related revisions have been updated, and additional statement has been added to the manuscript and cover letter. |

| EDCTP2 programme supported by the European Union (grant number RI2020S-3303 - GELA). The views and opinions of authors expressed herein do not necessarily state or reflect those of EDCTP. Please state what role the funders took in the study. If the funders had no role, please state: ""The funders had no role in study design, data collection and analysis, decision to publish, or preparation of the manuscript.""  If this statement is not correct you must amend it as needed.  Please include this amended Role of Funder statement in your cover letter; we will change the online submission form on your behalf |  |
| --- | --- |
| TYPOGRAPHICAL ERRORS  Pg 8: delete full stop at the end of the title Pg 14: (Statistical Analysis) correction “confident interval” for “confidence interval”  Pg 15: (Results) correction “summarized” for  “summarized” consistency in British spelling | All correctios have been made. |
| 3. Authors should read through the authors guideline and adhere strictly to it. | We have made adjustments accordingly. |
| 4. Authors should add more numbers to the statistical result of the abstract. | More results have been added to the abstract section and shown in red. |
| 5.Please remove the Table in the introduction | The table has been removed as requested. |
| 6.The statistical analysis did not describe a rigorous statistical analysis that is of quality for publishing in Plos one. I strongly recommend that authors should conduct bivariate analysis and if possible appropriate regression modelling to before the manuscript with be of high quality that can be published in PLOS ONE. The sample size is adequate for useful conclusion from the study | A bivariant analysis and multivariant analysis have been conducted and findings have been added to relevant sections. |

| Authors stated .".....standard deviation (SD), and range for continuous variables if normally distributed" What about if the continuous variable was not normally distributed? | This has been revised and reviewed in the manuscript. If not normally distributed interquartile ranges and medians have been used. |
| --- | --- |
| In the Result section, authors stated "Over a period of seven months 1066 participants were recruited for this study and had a recorded family planning method of their choice." From the methodology section, it appears that the authors described retrospective chart review of routinely collected data. Please authors should be explicit about what study was conducted. was it a retrospective or prospective study? | A prospective study was conducted. This has been revised in the manuscript. |
| Table 1 is not looking "professional" For example: "Delivery Characteristics" lumped many unrelated categories. One of the variables in that category is mode of delivery that can be vaginal delivery, operative vaginal delivery or caesarean section. Birth outcome such as live birth, fresh still birth, early neonatal death etc may be another category etc, | The tables have been split and edited as requested into table 2 and 3. |
| This study seems to be among postpartum women. When after delivery was the interview conducted.? If it was anytime post partum. It will be good to know how soon after delivery that women presented for family planning at the hospitals. | The data was collected from the medical records on the day of discharge after delivery. This has been stated in the methods section as well. |
| Table 2. gravidity and parity are not likely to be normally distributed. Thus median and IQR may be more appropriate. Please check | These statistics have been reanalysed and updated accordingly. |
| Discussion: Authors to cite relevant literatures as required | Revisions done. |
| Please ensure that DMPA had been previously defined in the manuscript | DMPA has been defined clearly in the text. |
| Are there any historical and structural issues that influences the contraceptive uptake pattern? | Additional paragraph added to discussion as requested. |
| Social cultural and economic determinants of health is a very important issue in South Africa. However, authors did not describe the ethnic characteristics of the studied participants. Occupation and income of the participants are also important information to provide. | This revision is addressed and discussed in the discussion section. |
| If the prevalence is so high, is it possible to collect further data for more months to assist to obtain more participants that will not take the contraceptive for easy comparative cross-sectional study? | This limitation is addressed in the limitations section. |
| What are the reasons for the comparatively high contraceptive uptake in this population as compared with other south African and global studies? | Revised and discussed in the discussion section. |
| Authors stated "There is also a theoretical risk that high dose progesterone exposure may impact the development of the infant brain. Authors should cite appropriate reference for this statement | The WHO statement that makes this claim is cited as well as the original data. |

All revisions and changes have also been marked in the text using red font and comment boxes.

Kind regards

Denise
