# Supplementary material for: Contraceptive uptake and characteristics in the immediate postpartum period among women attending Tygerberg Academic Hospital
Source: PLoS One. 2026 May 28;21(5):e0349790. doi: 10.1371/journal.pone.0349790 (PMC13218452; doi:10.1371/journal.pone.0349790)
Supplement: Attachment — Submitted filename: Response to Reviewers July 2025.docx [file pone.0349790.s004.docx]

15 July 2025

Dear Editor in Chief,

Thank you for the opportunity to re-submit our article to PLOS ONE journal. Please find below a rebuttal table outlining the changes and revisions requested for our submission.

| Changes requested | Revision action. |
| --- | --- |
| 1. Title: While I agree with Prevalence as being the main objective, `Sociodemographic risk and morbidity associated with contraception` was not studied, but rather as part of the eligibility criteria. I suggest that the title be reviewed. | Purposed new title: “**Contraceptive Uptake in the Postpartum Period: Prevalence Among Women Attending Tygerberg Academic Hospital.”** |
| 2. Aim: The aim, lines 73-78 should be rephrased. Line 78 seems presumptuous and and conclusive before the study. | This paragraph has been revised. See corrections and revisions in text. |
| 3. Methodology: The study cannot be cross-sectional and prospective at the same time as stated by the researchers. The data could as well have be collected retrospectively from January to July 2023. The methodology should be revised. The WHO eligibility criteria being a readily available document as referenced(Table 1) need not be included. | Thank you for your opinion. We have changed the study method to a descriptive study design to be more inclusive of the prospective data collection interval.  I have deleted the WHO table 1. All table numbers have been updated to follow sequence of numbers. |
| 4. Conclusions: This need rephrasing . Lines 341-348 cannot be deduced and recommendation made as such. | This paragraph has been revised. See corrections and revisions in text. |
| 5. Generally: Mixed British and American English abound in the write-up, eg Organization, recognised, individualized in lines 59, 51, 68 as examples. writer can crosscheck and provide uniformity. | Grammer, spelling formatting check have been done to entire manuscript. |

All revisions and changes have also been marked in the text using red font and comment boxes.

Kind regards

Denise Sproul
