# Supplementary material for: Contraceptive uptake and characteristics in the immediate postpartum period among women attending Tygerberg Academic Hospital
Source: PLoS One. 2026 May 28;21(5):e0349790. doi: 10.1371/journal.pone.0349790 (PMC13218452; doi:10.1371/journal.pone.0349790)
Supplement: Attachment — Submitted filename: Response to reviewers April 2026.docx [file pone.0349790.s006.docx]

**April 2026**

**Dear Editor in Chief,**

Thank you for the opportunity to re-submit our article to PLOS ONE journal. Please find below a rebuttal table outlining the changes and revisions requested for our submission.

| **Changes requested** | **Revision action.** |
| --- | --- |
| Removed “Prevalence” and edit to include characteristics. | Changed |
| Minor sentence structure alterations, grammar and punctuation requested through out paper. | All these requests have been made and are highlighted in red or strikethroughs. |
| Need to specify why such number was required. If you are going to explain it later, put something like "according to the method describe below". Or you may not put the number here. | Add in “according to the sample size calculation as described below,” |
| It is also not "prevalence" in the postpartum period because it lasts up to 6 weeks, so some women could have used FP within such period and the study did not collect that... | Changed to UPTAKE |
| It is very important to assess this percentage, because in most hospitals the uptake is usually lower. Is this because of the study the practitioners tried to convince women to the use, or is a well known place for "postpartum family planning (PPFP)? | This is addressed in the discussions section |
| This needs to be better described or add examples in parenthesis...you mean chemicals, asbestos, pesticies...etc., or you mean drugs... | Text changed to be clear, drugs. |
| What is this? Over 35? or what did you use? | Added “defined as older then of 35 years of age” |
| This needs to be better defined. They appear to be the Average (means) numbers... | Heading added, Mean (IQR) |
| What is this? | VL, means Viral Load. Edited. |
| Did you find the reason for not receiving it? (e.g., fear of husband, primiparous...) | We unfortunately did not interact with patients for this study, we reviewed the discharged and medical records on the day of discharge to collect data. There was no documentation of the reason for declining. |
| You have to describe each category, if you are going to use them (e.g., Category 1: No restriction on use.  Category 2: Advantages generally outweigh risks.  Category 3: Risks generally outweigh advantages.  Category 4: Unacceptable health risk (method should not be used). | I had previously had a table for this description, and a previous reviewer commented to take it out as it is common knowledge. I have reinserted a figure for reference.  Line 183 (figure 2) |
| DMPA? Line 192 | Addressed, depo medroxyprogesterone acetate, abbreviation showed first in line 177 |
| ??? [Chronic Hypertension? - hast to be put in full before first use of acronyms] line 192 | Addressed, Chronic Hypertension, abbreviated in table 1 in line 165 |
| Again, interpreting it is usually that lower parity women could know more about FP, perhaps are less confident that husbands will like their use of FP, etc., so they prefer a method that is not "seen" at home...go look for that literature...(usually qualitative studies). | Thank you for the opinion, addressed these issues in the discussion sections. |
| This is NOT TRUE. DMPA effectiveness is very high but, like any method, it depends on how often/on time the users continue to use them. See summary: DMPA injection ("Depo-Provera) has a high, yet user-dependent, effectiveness, with a first-year failure rate of roughly 0.2–0.3% (per 1,000 users) with perfect use. In typical, real-world use, this rate increases to 3-6% due to delayed or missed injections, which are generally recommended every 12–13 weeks."). | Sentence deleted |
| Yes, but at immediate postpartum period the uterus is too large, so potential users have to come back to use IUCD after a few weeks, thus several don't come back | Added in context line 270 |
| 3% is not "majority"! How can you put this?...the majority in Cat III it was followed by Cat I (24.2%) and Cat II (22.5%), Rephrase to understand better... | Rephrased |
| Please also read the reflection where we put that it's also usually younger women who may be afraid of the husband reaction, thus a 3-month injectable is convenient for them... | Thank you for this suggestion. In our setting majority of younger women are single and this statement would not be applicable to them. |

All revisions and changes have also been marked in the text using red font and comment boxes.

Kind regards

Denise Sproul
